# Epidemiological changes in the pattern of children’s traumatic injuries at Hong Kong emergency departments during the COVID-19 pandemic: a retrospective, single-institutional, serial and comparative study

**DOI:** 10.1007/s00383-024-05772-3

**Published:** 2024-07-16

**Authors:** Jaime Tsz-wing Tsang, Adrian Chi-heng Fung, Heidi Hay-tai Wong, Wing Chiu Dai, Kenneth Kak-yuen Wong

**Affiliations:** 1 Department of Surgery, School of Clinical Medicine, The University of Hong Kong, Hong Kong, China; 2 Trauma Service, Queen Mary Hospital, Hong Kong, China

**Keywords:** Paediatric trauma, Epidemiology, COVID-19 pandemic, Children

## Abstract

**Introduction:**

Trauma is the leading cause of paediatric mortality and morbidity. Stay-home regulations for coronavirus disease 2019 (COVID-19) reportedly changed trauma severity, yet data from Hong Kong were lacking. This study examined Hong Kong’s spectrum of paediatric trauma and addressed knowledge gaps concerning epidemiological changes during COVID-19.

**Methods:**

Children with traumatic injuries who attended a tertiary trauma centre from January 2010 to March 2022 were included in this retrospective, cross-sectional study. We analysed demographic and clinical data and conducted unadjusted bivariate analyses of injury patterns before and after the pandemic.

**Results:**

In total, 725 children attended the Accident and Emergency Department due to trauma, 585 before and 140 during COVID-19. The male-to-female ratio was 1.84:1. The 90-day trauma-related mortality was 0.7%. The overall Injury Severity Score was 3.52 ± 5.95. The paediatric trauma incidence was similar before and after social-distancing policies (both 5.8 cases monthly). Gender, ISS distribution, intensive care unit stay length, and hospital stay length values were similar (*p* > 0.05). Trauma call activation (8.4% vs. 5.7%, *p* = 0.002) and road traffic accidents (10.6% vs. 5.7%, *p* = 0.009) significantly decreased, yet younger-patient injuries (< 10 years old; 85.7% vs. 71%, *p* < 0.001), burns (28% vs. 45.7%, *p* < 0.001), and domestic injuries (65.5% vs. 85.7%, *p* < 0.001) significantly increased. No significant self-harm, assault, or abuse increases were found.

**Conclusions:**

The paediatric trauma incidences were similar before and during the pandemic. However, domestic and burn injuries significantly increased, highlighting the importance of injury prevention.

## Introduction

On 11 March 2020, the World Health Organization (WHO) declared coronavirus disease 2019 (COVID-19) a global pandemic. Public health interventions, including the issuance of quarantine orders or stay-home regulations, were implemented worldwide to dampen viral transmission [1]. Alongside resulting morbidity, mortality, and economic losses, COVID-19 also significantly influenced social behaviour, mental well-being, and health service utilisation worldwide through its associated social-distancing policies.

While trauma is the leading cause of death and major morbidity among children and adolescents worldwide, reportedly, COVID-19 stay-home regulations changed the volume, pattern, and severity of trauma in the paediatric population. Quarantines were associated with a 48% reduction in paediatric trauma in New Zealand [2]. A similar decline in overall trauma encounters and emergency department visits was reported in part of the USA after lockdown policies were enforced; however, in places such as Los Angeles, paediatric trauma activations did not differ during the pandemic [3–5]. On the other hand, conflicting evidence was obtained concerning COVID-19 stay-home policies’ impact on trauma severity and mechanisms, including variable epidemiological changes in burn injuries, home accidents, and motor vehicle accidents, as well as the occurrences of abuse, assault, self-harm, and domestic violence [6].

In Hong Kong, social-distancing policies including the Prevention and Control of Disease Regulation (Cap. 599G), which restricted the number of people in group gatherings at public places, as well as a school suspension ordered by the Education Bureau were first imposed in February 2020[7]. Paediatric fractures in Hong Kong reportedly declined during the pandemic [8]. However, data on these regulations’ potential effects on other aspects (for instance, road traffic accidents, burn injuries, etc.) of paediatric trauma’s occurrence, pattern and severity in Asian cities are lacking.

Since COVID-19’s disease burden continues to fluctuate, and the pandemic could recur, with communities inevitably returning to quarantine measures, heightening the awareness of changes in the local paediatric injuries’ patterns and severity is worthwhile so that relevant measures can be undertaken. Accordingly, the current study examined the spectrum of paediatric trauma in Hong Kong during COVID-19 and addressed knowledge gaps concerning its epidemiological changes.

## Methods

### Study design

This retrospective, single-centre, comparative study was conducted through the prospectively collected trauma registry of a tertiary paediatric trauma centre concerning children who attended an emergency department with various degrees of trauma. This retrospective cohort study was reported according to the Strengthening the Reporting of Observational Studies in Epidemiology (STROBE) guidelines for observational studies when appropriate [9]. The study was also reviewed and approved by the hospital’s institutional review board (reference number: UW 24-288).

### Setting, patients, and data collection

Our centre is a tertiary referral centre for the specialist services of a network of hospitals covering Hong Kong Island and Kowloon. It is considered one of two key designated trauma centres on Hong Kong Island [10]. In general, trauma patients are either directly admitted from the scene of their injury to our centre’s emergency department or undergo secondary trauma diversion from a hospital in our network [11]. The network serves a population of about 0.6 million people.

Data were retrieved from our centre’s prospectively collected, standardised trauma registry [10]. The registry data represent information from most of the population that experienced trauma of one of the major hospital networks in Hong Kong since the establishment of prehospital trauma diversion and secondary trauma diversion in 2006. The registry data set comprises an inpatient cohort and emergency attendance analysis. Chart reviews were conducted by a trained trauma coordinator to identify the majority of the data in the core set and extract supplementary data from electronic patient record system concerning details of traffic-, self-harm- and assault-related injuries, etc. Anonymised data were then entered into the registry by trained trauma registrars using standardised methods [10, 12].

A retrospective review of patient demographics, injuries’ type and mechanism, and clinical outcomes was conducted using data from the trauma registry from 1 January 2010 to 31 March 2022. We included all children under 18 years of age in the registry. The data in our analysis comprised patient demographics (including gender and age), hospital-processing information, classified activities, the need for trauma call activation, the types and severity of sustained trauma (defined using Injury Severity Scores [ISSs]), trauma mechanisms and the environments where injuries occurred.

### Outcome measures

The study’s primary outcomes were the incidence of trauma and paediatric trauma call activation. The secondary outcomes were selected from the registry’s demographic and clinical data, including the types and severity of sustained injuries, injuries’ environments, paediatric intensive care unit stay lengths and overall hospital stay lengths, the procedures performed for operative patients, the sustained morbidities and mortality. We defined the pre-pandemic period as 1 January 2010 to 31 January 2020 and the pandemic period as 1 February 2020 to 31 March 2022, based on Hong Kon’s social-distancing regulations and school suspensions that began on 1 February 2020.

### Statistical methods

All data were statistically analysed and compared. The study’s statistical analyses were performed using the Statistical Package for the Social Sciences (SPSS; IBM, USA), Version 26. Descriptive statistics were presented as the number of units (*n*) and percentages (%). Data were expressed as means and standard deviations. Continuous variables were analysed using Student’s *t* test and an analysis of variance (ANOVA) test, as appropriate. Ordinal variables were analysed using the Mann–Whitney *U* test, and categorical variables were analysed using the Chi-square test. An unadjusted bivariate analysis of the injury patterns between the pre-pandemic and pandemic periods was performed. A *p* value < 0.05 was considered statistically significant.

## Results

### Study sample characteristics

In total, 725 children attended the Accident and Emergency Department (AED) due to trauma during the study period, 585 before COVID-19 (1 January 2010–31 January 2019) versus 140 during COVID-19 (1 February 2019–31 March 2022. The study included 585 boys and 140 girls. Thus, overall, boys were more commonly injured, with a male-to-female ratio of 1.84:1. However, no significant difference in gender ratios was observed between the pre-COVID-19 and COVID-19 periods (1.8:1 vs. 1.9:1, *p* = 0.807). Of the included patients, 538 were 10 years old or younger, while 187 were 11 years old or older. Thus, a larger proportion of younger children sustained injuries. Injuries involving patients 10 years old or younger increased significantly from before COVID-19 (71%) to during COVID-19 (85.7%; *p* = 0.001). The patients’ mean age was significantly lower during COVID-19 (6.5 ± 5.4 vs. 4.6 ± 4.3, *p* < 0.001; Table 1).

**Table 1:** Patient and clinical characteristics, stratified by pre-COVID-19 and COVID-19 periods

	Before COVID-19 (*n* = 585)	COVID-19 (*n* = 140)	*p*
Occurrence of trauma (cases/month)	5.8	5.8	/
Gender (M:F)	1.8:1	1.9:1	0.807
Age group (years)			**0.001**
0–10	418 (71.5%)	120 (85.7%)	
11–18	167 (28.5%)	20 (14.3%)	
Age (mean ± SD)	6.5 ± 5.4	4.6 ± 4.3	** < 0.001**
Trauma call activation	49 (8.4%)	8 (5.7%)	**0.009**
Injury Severity Score	3.5 ± 6.1	3.4 ± 4.9	0.828
Mechanism of injury			0.500
Blunt	577 (98.6%)	137 (97.9%)	
Penetrating	8 (1.4%)	2.1 (3%)	
Child abuse-related injury	16 (2.7%)	4 (2.9%)	0.924
Length of hospital stay (days)	8.2 ± 14	9.8 ± 10	0.216
Length of ICU stay (days)	0.6 ± 3.1	0.5 ± 1.9	0.895
Mortality	5 (0.9%)	0	0.356

Statistically significant values are denoted in bold

### Hospital stays’ incidence, severity, and length

The overall incidences of trauma among paediatric patients who attended the emergency department were similar before and after COVID-19 social-distancing policies, with 5.8 cases per month for both periods. Trauma call activation occurred for 7.8% of patients (*n* = 57). A significant reduction in the number of trauma call activations was observed (8.4% vs. 5.7%, *p* = 0.002). Moreover, the 90-day trauma-related mortality was 0.7%, and one patient died within 24 h of admission. There was no significant difference between the two time periods (pre-COVID-19 0.9% vs. COVID-19 0%, *p* = 0.356). The overall ISS was 3.52 ± 5.95, and it appeared to increase with age (*ρ* = 0.289, *p* < 0.01). The ISSs during the pre-COVID-19 period (3.5 ± 6.1) and the COVID-19 period (3.4 ± 4.9) did not differ significantly (*p* = 0.828). Hospital stay lengths (8.2 ± 14 vs. 9.8 ± 10, *p* = 0.216) and paediatric ICU stay lengths (0.6 ± 3.1 vs. 0.5 ± 1.9, *p* = 0.895) in days did not significantly differ either (Table 1).

### Injury mechanisms and patterns

**Fig. 1:**
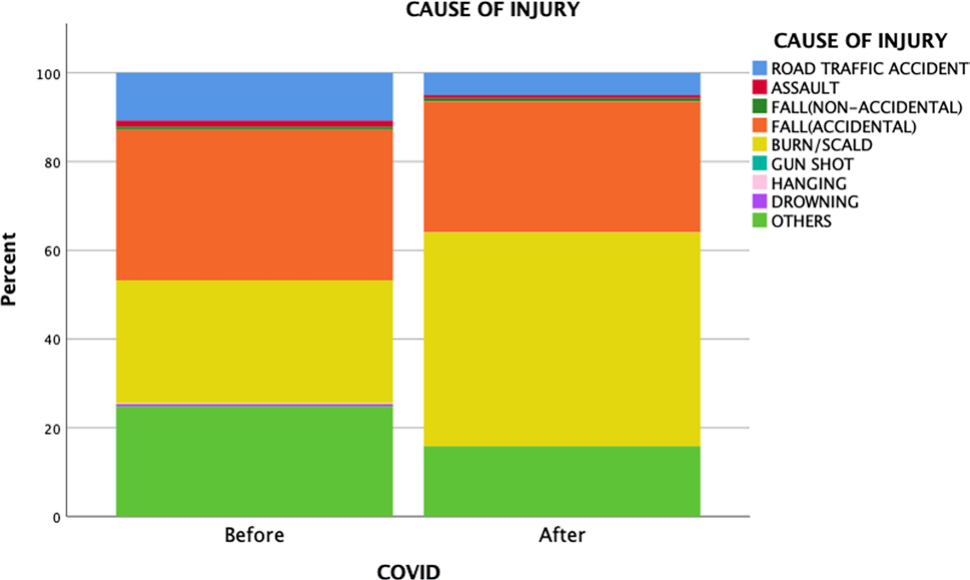
Changes in cause of injury before and after the COVID-19 pandemic

**Fig. 2:**
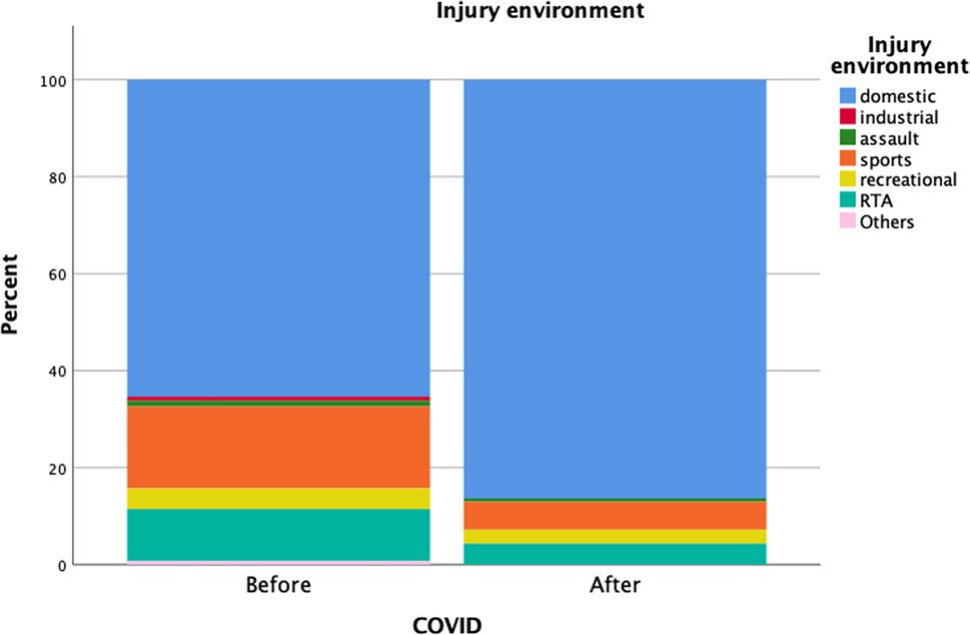
Changes in injury environment before and after the COVID-19 pandemic

An examination of the patients’ mechanisms of injury revealed similar proportions of blunt injuries (98.6% vs. 97.9%) and penetrating injuries (1.4% vs. 3%) before and after COVID-19 (*p* = 0.5). Injury pattern details are presented in Table 2. Example of blunt trauma included cerebral concussion as a result of road traffic accident, subdural haematoma as a result of fall, fracture of extremities as a result of road traffic accident and sports injuries, splenic and liver lacerations after motor vehicle accidents, fall with torn frenulum of lip, etc. For penetrating injuries, examples in the cohort included forearm injury by broken parts of a chair, stab injury of thigh by an army knife, floor of mouth penetration by the hook of a coat hanger, etc. “Miscellaneous” causes of injury encompassed heterogenous group of unclassifiable injury mechanisms including injury by broken plastic cup while drinking water, injury by falling broken glass, injury of limbs while running, finger amputation by accidentally closing of cupboards against own fingers, electric shock injury, chin laceration by badminton racket when playing with siblings, etc. As for the 229 patients with burn, the mean total body surface area of burn (TBSA) was 6 ± 5%. Patients with TBSA 0–10% and 11–20% accounted for 92.6% (*n* = 212) and 4.8% (*n* = 11), respectively. There were six severe burn patient with TBSA > 20%. Majority of the burn were resulted from hot water burn (63%, *n* = 144), other causes included fire burn, electrical burn, caustic burn, and burn from other fluids e.g. soup, congee, tea, etc. There was not any inhalation injury in our cohort of patients. Significantly fewer road traffic accidents (RTAs; 10.6% vs. 5.7%, *p* = 0.009) and fewer sports-related injuries (16.9% vs. 5.7%, *p* < 0.001) occurred during the pandemic. Conversely, burn injuries (28% vs. 45.7% *p* < 0.001) increased during the pandemic. However, there was no significant difference in the severity of burn before and during the COVID-19 pandemic (TBSA 6 ± 5% vs 5 ± 3%, *p* = 0.305). Furthermore, a higher proportion of injuries occurred in domestic environments (65.5% vs. 85.7%, *p* < 0.001) during the pandemic (Figs. 1 and 2). No significant increases in self-harm (0.7% vs. 0, *p* = 0.328), assault (1.4% vs. 0.7%, *p* = 0.53), or abuse (2.7% vs. 2.9%, *p* = 0.924) were found during the pandemic.

**Table 2:** Mechanisms and patterns of injury, stratified by pre-COVID-19 and COVID-19 periods

	Before COVID-19 (*n* = 585)	COVID-19 (*n* = 140)	*p*
Mechanisms of injury			0.500
Blunt	577 (98.6%)	137 (97.9%)	
Penetrating	8 (1.4%)	2.1 (3%)	
Causes of injury			
Road traffic accident	62 (10.6%)	8 (5.7%)	**0.009**
Fall	200 (34.3%)	44 (31.4%)	0.53
Burn	165 (28.3%)	64 (45.7%)	** < 0.001**
Hanging	1 (0.2%)	0	0.27
Drowning	3 (0.5%)	0	0.78
Assault	8 (1.4%)	1 (0.7%)	0.53
Miscellaneous	144 (24.7%)	23 (16.4%)	0.068
Environment of injury			
Domestic	383 (65.5%)	120 (85.7%)	** < 0.001**
Industrial	5 (0.9%)	0	0.86
Sports related	99 (16.9%)	8 (5.7%)	** < 0.001**
Recreational	25 (4.3%)	4 (2.9%)	0.44
Traffics	62 (10.6%)	8 (5.7%)	**0.009**
Miscellaneous	11 (1.8%)	0	0.33
Self-harm-related injury	4 (0.7%)	0	0.328
Child abuse-related injury	16 (2.7%)	4 (2.9%)	0.924

Statistically significant values are denoted in bold

## Discussion

While the COVID-19 pandemic led to a significant decline in social interactions through stay-home policies, our study found that trauma cannot be avoided via quarantines, and no significant differences in overall paediatric trauma incidences were observed during the pandemic. This finding seems to differ from the findings of other studies that have shown reductions in trauma encounters [2–4]. However, consistent with worldwide reports, our study revealed a significant reduction in trauma call activations after social-distancing policies’ implementation. This result might be attributed to the reduction in RTAs (which usually cause more severe injuries than other mechanisms) as a result of reduced motor vehicle travel due to social distancing. Moreover, children’s adoption of more sedentary lifestyles due to home confinement during the pandemic led to a significant decline in sports activities among this population [13]. This change invariably led to fewer severe sports-related injuries and, thus, the reduction of trauma call activations.

In 2021, Chaudhari et al. reported no significant differences in median ISSs, the need for hospitalisation, ICU rates or hospital stay lengths between the pre-pandemic and pandemic periods [5]. Sanford et al. reported a similar finding in their retrospective study published during the same year [4] (Table 3). Our cohort study corroborates the results of these previous publications and demonstrates no significant difference in ISS distribution or hospital and ICU stay lengths. However, in 2021, Bessoff et al. conversely reported higher ISSs, more severe injuries, and longer ICU stays among patients who presented to a hospital during the pandemic [14]. Nonetheless, this finding might be attributed to the increasing amount of penetration trauma and firearms in homes, which are not entirely applicable to our local context since ammunition and arms possession is illegal in Hong Kong without proper licences.

Echoing many other studies worldwide, we observed a significant increase in burn injuries during the pandemic in Hong Kong [4, 15]. Alongside this increase in incidences, several studies have shown increases in the severity, total body surface area (TBSA) and ICU admissions of burns during the pandemic [16–18]. However in our cohort, no significant difference was demonstrated in the extent of burn during the pandemic. Moreover, they also demonstrated an increased prevalence of house fires and scalding injuries, which echoes our finding of increased injuries in domestic settings. The postulated reasons for increased domestic injuries include the loss of social connections and family support, the lack of school support, the reduced supervision of children due to adults’ tendency to work from home, the lack of structured childcare environments, and inevitable variance in childcare and parental monitoring. Our findings indicate that further measures for burn prevention and management, such as public education, are warranted to ensure children’s safety during the pandemic. Furthermore, policymakers should note that burn facilities must not be reduced despite limited health services as a result of the COVID-19 pandemic. Indeed, such facilities should perhaps be enhanced, given the escalated incidence of burn injuries (see Table 3).

**Table 3:** Comparison of change of trauma incidence and pattern reported in other studies

Articles	Significant findings	Category	Before COVID-19 mean/median (IQR)/*n*(%)	COVID-19 mean/median (IQR)/*n*(%)		*p*	Remarks
Bessoff, K.E., et al. epidemiology of pediatric trauma during the COVID-19 pandemic shelter in place. Surg open sci, 2021. 6: p. 5–9	Mechanisms of injury	(1) Gunshot	71 (1.8%)	36 (3.3%)	↓	0.038	
		(2) Non-motorized vehicles	360 (8.9%)	163 (15%)	↓	< 0.01	
	Trauma volume 16 days after implementation of shelter-in-place	–	14.2%	5.7%	↓	–	
	Median Injury Severity Score	–	4 (4,9)	5 (4,10)	↑	< 0.01	
	Severe injuries (ISS > 25)	–	3.5%	5.8%	↑	0.019	
Sanford, E.L., et al. changes in pediatric trauma during COVID-19 stay-at-home epoch at a tertiary pediatric hospital. J pediatr surg, 2021. 56(5): p. 918–922	Mechanisms of injury	Burn	13.6%	20.2%	↑	< 0.001	No significant findings in Injury Severity Score, ICU rates
		Penetrating trauma	7.2%	11%	↑	0.002	
		Blunt trauma	79.3%	68.9%	↓	< 0.001	
		Motor vehicle crashes	53.2%	27%	↓	< 0.001	
		Automobile	50%	14%	↓	< 0.001	
		Falls	52.2%	36%	↓	< 0.001	
		Sports related	40.2%	8%	↓	< 0.001	
	Trauma occurrence	–	460	392	↓	< 0.001	
Chaudhari, P.P., et al., epidemiology of paediatric trauma during the coronavirus disease 2019 pandemic. J pediatr surg, 2022. 57(2): p. 284–290	Mechanisms of injury	Motor vehicle injury	367 (13.6%)	269 (15.8%)	↑	0.044	No significant findings in paediatric trauma activation, Injury Severity Score, ICU rates
		Assault	105 (3.9%)	42 (2.5%)	↓	0.01	
		Gunshot	202 (7.5%)	178 (10.5%)	↑	< 0.001	
		Thermal	31 (1.2%)	52 (3.1%)	↑	< 0.001	
		Sports and recreation	152 (5.6%)	25 (1.5%)	↓	< 0.001	
	Intubated	–	135 (11.6%)	58 (6.8%)	↓	< 0.001	

Our study also revealed that younger children were more easily injured during the pandemic. This finding might be explained by such children’s vulnerability and youth, given the lack of family and school support during the pandemic. In their large-scale, cross-sectional population study on vulnerable groups of children during the COVID-19 pandemic, Tso et al. reported that the risk of psychosocial problems was higher among children with special educational needs or acute or chronic disease, mothers with mental illnesses, single-parent families, and low-income families [19]. Increased injuries from unusual foreign bodies were reported in children as a result of psychosocial issues that arose from home confinement measures [7]. Identifying such vulnerable groups of children and families to offer parental education, home visits and modifications that support their needs is imperative. Despite widespread concerns of the public about the potential increase in the incidence of abuse, assaults and self-harm during the pandemic, our work did not note any significant differences between the pre-COVID-19 and COVID-19 periods. This result contrasts with the findings of studies published in the United States that exhibited a twofold increase in non-accidental trauma (NAT) [20, 21]. Similar findings were also presented in studies from China, Brazil, France, and Italy [22–24]. Although our results aligned with some works from the UK and Ireland which showed no observable increase in NATs, drafting strategies to mitigate this possible secondary effect of social distancing is crucial. The major reason for the above is that unchanged or even decreased NATs during the pandemic may have resulted from fewer opportunities for detection, rather than an actual decrease in incidences. The suspension of schools, as key community partners in detecting and reporting abuse, might have led to a seemingly reduced rate. Meanwhile, a study conducted in Germany confirmed that parental stress during the pandemic was significantly correlated with child maltreatment, whether verbal or physical abuse [25]. Social-distancing regulations might have raised parental stress since some parents might have had to work from home while caring for their children. Additionally, reduced socialising, a possible stress reliever, could have contributed to children’s maltreatment. Therefore, despite our study’s results, close observations of NAT trends remain imperative.

Overall, our findings may help guide injury or trauma prevention campaigns and measures for future pandemic surges. For instance, household education concerning the vigilant use and positioning of kitchenware, home preventive measures and management for scalding and burn injuries, or even home modification strategies, may be strengthened to prevent younger children from sustaining burn injuries during quarantines. Meanwhile, social-distancing policies must be coupled with increased social support and a transparent, easily accessible reporting system to discourage violence. Public education on mental health and psychological support for both adults and children should also be strengthened to improve coping and possibly reduce non-accidental injuries or children’s maltreatment.

Despite our findings’ applicability, our study was conducted retrospectively at a tertiary trauma and burn centre in Hong Kong, so its generalisability might be limited. Demographic factors such as patients’ geographical locations, socioeconomic status and parental education levels were not studied. Moreover, the number of trauma patients included in this study largely depended on whether such patients had presented at the AED; some children who experienced trauma, especially less severe trauma, may not have presented due to the avoidance of contracting COVID-19 during unnecessary hospital stays. Therefore, the actual number of paediatric trauma incidences could be higher than stated in the current study. In Hong Kong, though social-distancing regulations including Cap. 599G and school suspensions were implemented in 2019, the Centre for Health Protection launched the ‘Stay Hong Safe’ scheme in February 2022. The scheme arranged for COVID-19 close contacts and household contacts who were deemed appropriate candidates via assessments to undergo home quarantines for 14 days and 4 days, respectively. Related citizens had to stay in their dwellings and wear electronic wristbands throughout the quarantine period and undergo regular monitoring of their physical conditions. This scheme might have further affected the pattern of paediatric trauma, which was not studied in the current work.

Despite limitations, to our understanding, this study has provided the first thorough analysis of local social-distancing policies’ effects on the incidence, severity, and patterns of paediatric trauma in an Asian city. Imperatively, healthcare professionals must be well prepared for changes in trauma patterns. At the same time, this study might serve as a reference for health policy enactment and resource or expenditure allocation, since public health policies should be adjusted to align with social-distancing policies’ effects to ensure children’s safety during a pandemic.

## Conclusion

Overall, our study identified similar incidences of paediatric trauma before and during the pandemic period. However, a significant increase in domestic and burn injuries was observed among younger children during COVID-19. These findings highlight important epidemiological changes and injury prevention measures that should be undertaken during future pandemics.

## Data Availability

No datasets were generated or analysed during the current study.
